# Building evidence‐based interventions to improve staff well‐being in paediatric critical care using the behaviour change wheel

**DOI:** 10.1111/nicc.13228

**Published:** 2025-01-08

**Authors:** Rachel L. Shaw, Isabelle Butcher, Sarah Webb, Heather P. Duncan, Rachael Morrison

**Affiliations:** ^1^ Institute of Health & Neurodevelopment, College of Health & Life Sciences Aston University Birmingham UK; ^2^ Department of Psychiatry University of Oxford Oxford UK; ^3^ Paediatric Critical Care Birmingham Children's Hospital Birmingham UK

**Keywords:** critical care, health personnel, intervention, paediatricswell‐being

## Abstract

**Background:**

Research has demonstrated that staff working in Paediatric Critical Care (PCC) experience high levels of burnout, post‐traumatic stress and moral distress. There is very little evidence of how this problem could be addressed.

**Aim:**

To develop evidence‐based, psychologically informed interventions designed to improve PCC staff well‐being that can be feasibility tested on a large scale.

**Study Design:**

The Behaviour Change Wheel (BCW) framework guided systematic development of the interventions. This process was informed by a review of existing well‐being initiatives and a survey of PCC staff's awareness and uptake of initiatives identified.

**Results:**

Together with empirical evidence, the BCW process produced two bespoke ‘SWell’ (*S*taff *Well*being) interventions tailored for delivery in UK PCC units. The two group‐based interventions, *Mad‐Sad‐Glad* and *Wellbeing Images* involve the Behaviour Change Techniques (BCTs) of *self‐belief*, *social support*, *feedback and monitoring*. These BCTs align closely with the psychological concepts of self‐efficacy, self‐regulation and the psychological theory of how to thrive.

**Conclusions:**

Tailored, evidence‐based, psychologically informed SWell (*S*taff *Well*being) interventions are likely to be feasible and have the potential of making significant differences to individual staff members and the PCC workforce as a whole. Associated investments in the psychological health of the workforce and time to prioritize well‐being interventions are required for change to occur and be maintained.

**Relevance to Clinical Practice:**

The SWell (*S*taff *Well*being) interventions could impact directly on the well‐being of PCC staff and their ability to thrive in the workplace. Indirectly, they could reduce staff attrition, sickness absence and improve patients' and families' experiences of care.

**What is known about the topic**

- Levels of burnout, post‐traumatic stress and moral distress are higher among Paediatric Critical Care (PCC) staff than in other health care staff.
- PCC units are high‐pressure environments in which delivering staff interventions is challenging.
- There is very little research to suggest what interventions may work to improve PCC staff well‐being.

**What this paper adds**

- We have used a systematic framework—the Behaviour Change Wheel—novel to critical care nursing, to develop an evidence‐based and psychologically informed bespoke SWell (*S*taff *Well*being) intervention package to improve PCC staff well‐being.
- The Behaviour Change Wheel ensures intervention development is transparent, but also that it meets the needs of the target population and environment.
- The SWell (*S*taff *Well*being) interventions designed and produced as part of this project are likely to prove feasible to deliver in busy PCC units and hold promise to produce a positive impact on PCC staff well‐being.

## 1 INTRODUCTION AND BACKGROUND

Paediatric critical care (PCC) is a highly stimulating, yet high‐pressure work environment, which can have a negative psychological impact on staff.^1^, ^2^ Not only are staff required to deliver complex, life‐saving care, but they need to develop compassionate relationships with patients' parents during some of their most traumatic experiences.^3^ The UK National Health Service (NHS) Staff Survey 2023 (an independent annual survey, which includes staff from PCC units) found 41.71% of NHS staff reported feeling unwell due to work‐related stress, 32.40% said there were sufficient staff for them to do their job properly, and while there have been very slight improvements in overall burnout scores since 2022, 42.73% said they felt worn out at the end of their shift and 31.18% said they found their work emotionally exhausting.^4^

Worldwide evidence has consistently shown that staff working in PCC have higher levels of burnout, post‐traumatic stress and moral distress than other health care staff groups.^2^, ^5^, ^6^ A recent UK survey of PCC staff (*n* = 1656) used the Moral Distress Scale‐Revised^7^ to determine that 36% of nurses, 18% of physicians and 20% of other staff scored above threshold for moral distress; the Maslach Burnout Inventory^8^ identified 50% of nurses, 37% of physicians and 45% of other staff had high burnout scores; and the Trauma Screening Questionnaire^9^ identified 31% of nurses, 16% of physicians and 11% of other staff experienced symptoms of post‐traumatic stress.^2^ Evidence shows that burnout, moral distress and excessive workload are the most significant predictors of work‐related stress and intentions to leave the profession.^10^, ^11^ Put this alongside a national crisis in NHS staff shortages and there is a considerable workforce problem.^12^

Despite recognition within the profession, represented by the paediatric critical care society (PCCS)^13^ and the UK Committee of Health and Social Care^14^ that staff workload and burnout are significant problems, there is very little in terms of evidence‐based solutions. Research examining coping strategies has failed to identify successful techniques for improving PCC staff well‐being.^5^, ^15^

Perhaps this is not surprising because well‐being is difficult to define in terms other than a state of ‘balance’^16^ or ‘the state of being comfortable, happy or healthy’.^17^ Furthermore, there is a paucity of research examining PCC staff's own perceptions of workplace well‐being. A notable exception is an Australian study^18^ in which PCC staff identified threats to their well‐being, including: feeling under‐prepared for the role, distress associated with ‘lingering’ cases, non‐accidental injuries and the feeling of isolation due to being unable to share their distress with others. Protective factors included: finding the work stimulating and meaningful, belonging to a team and using ‘black’ humour as armour. The authors supported previous calls^19^, ^20^ for research to fill the current gap by developing interventions to mitigate risk factors and build protective factors for well‐being to enable staff to deal with the coexistence of a deep sense of work satisfaction alongside the psychological distress often experienced.^18^ It is clear from the evidence that eliminating psychological distress completely will not be possible; rather what is required are interventions to create a psychologically safe workplace, which prioritizes staff well‐being.^21^ This requires multilevel interventions—at the systemic and individual levels, to build a psychologically safe environment through education and well‐being policies, provision of (peer) support, mentorship and psychological therapies when required.^22^

This study was conducted to develop an evidence‐based and theoretically informed intervention to improve PCC staff well‐being. At the outset, it was uncertain what form the intervention would take (individual, group‐based, peer‐led, specialist‐led, online, in person). However, the evidence was clear that it was necessary to take positive action and focus on understanding how to improve PCC *staff well‐being* instead of measuring psychopathology. To achieve this, exploratory work was required to first understand what well‐being means to PCC staff and second to examine the kinds of initiatives PCC units have developed as part of their attempts to boost the well‐being of staff. These findings were then used to inform the development of staff well‐being interventions. The interventions were named ‘*SWell’*, standing for *S*(taff) *Well*(being). They were developed using a framework, the behaviour change wheel (BCW),^23^ which is recommended by Public Health England^24^ to guide the development of interventions to change behaviour and improve health, and which aligns with the National Institute of Health Research's guidance for developing complex interventions.^25^ In short, this paper reports the process of building the *SWell* interventions; testing feasibility and effectiveness is out of the scope of this paper.

### 1.1 Research objectives

1. To review existing well‐being initiatives in current use in a UK PCC unit.
2. To produce staff well‐being interventions that can be tested for feasibility in UK PCC units.

### 1.2 Research questions

- What well‐being initiatives are currently being offered to PCC staff?
- What Behaviour Change Techniques (BCTs), or active ingredients, are likely to produce successful staff well‐being interventions?
- What is likely to be the most appropriate mode of delivery for staff well‐being interventions in the PCC setting?

## 2 DESIGN AND METHODS

### 2.1 Design

To achieve these objectives, existing well‐being initiatives being delivered in one UK PCC unit were surveyed to help identify likely successful BCTs. Behaviour Change Techniques are ‘active ingredients’ extracted from systematic reviews of behaviour change interventions which have been collated into a taxonomy for use in the development of new interventions.^26^ Using BCTs ensures transparency of ‘active ingredients’ in a new intervention which is fundamental for researchers and practitioners to identify which elements of an intervention make it work.

**FIGURE 1:**
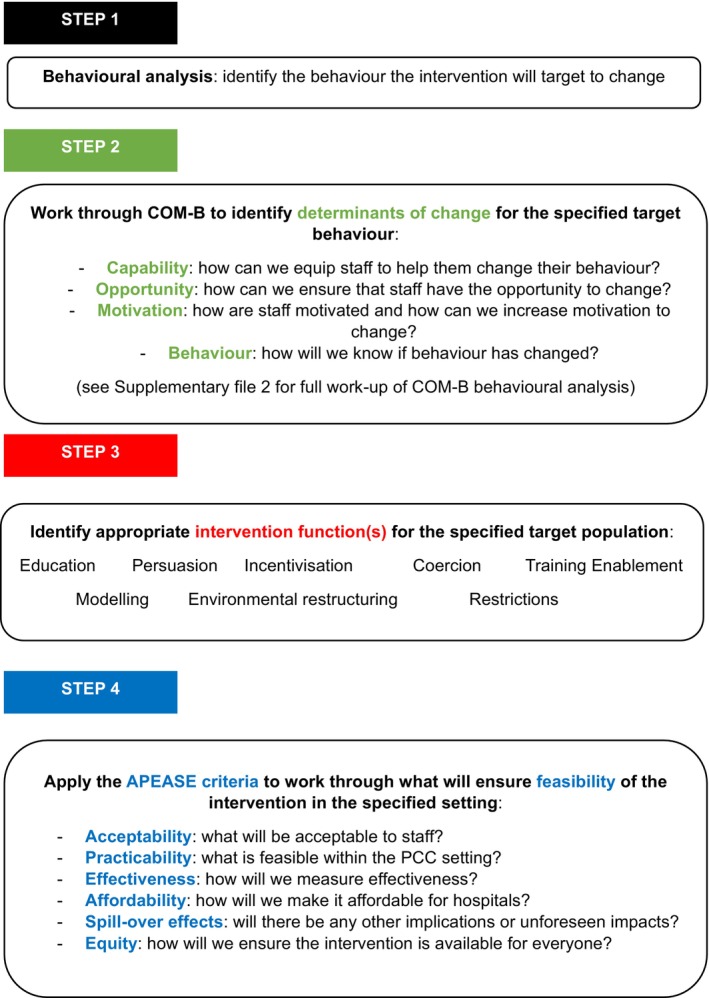
Steps within the behaviour change wheel intervention development framework.

The SWell interventions were then built using the BCW,^23^ which is a systematic intervention development framework for building behaviour change interventions in a way that is informed by behavioural theory and previous evidence. The theory used is known as the Capability‐Opportunity‐Motivation model of Behaviour, or COM‐B, which identifies those three factors—capability, opportunity, motivation—as determinants of behaviour change.^23^, ^27^ The BCW framework involves four steps: (i) *Behavioural analysis*—identification of the behaviour the intervention will target to change, (ii) *COM‐B determinants of change*—identifying how capability, opportunity and motivation for that behaviour change can be produced by the intervention, (iii) *Intervention functions*—identifying the appropriate functions used to change behaviour, (iv) *APEASE feasibility testing*—using what is known as the APEASE criteria to assess feasibility of the intervention (by exploring *A*cceptability, *P*racticability, *E*ffectiveness, *A*ffordability, *S*pillover effects and *E*quity). The BCW steps are outlined in Figure 1.

### 2.2 Sample and setting

This study took place in a large PCC unit in England (The PCC unit in this study caters for 35 specialties, including respiratory, cardiac, liver, general surgery, ear nose and throat (ENT), spinal, orthopaedics, metabolic, endocrine, neurology and neurosurgery. As the designated trauma centre in the region, it has access to all the radiological, surgical and acute care specialties needed to provide care for a child who has undergone trauma. It is commissioned to provide Extra Corporeal Membranous Oxygenation (ECMO) for respiratory and heart problems). Two purposive samples were established: (i) senior clinical staff and education team members were invited to take part in a review of existing well‐being initiatives on the unit; (ii) mixed‐discipline clinical staff employed in PCC were invited to take part in a survey gathering information about the awareness and uptake of well‐being initiatives identified.

### 2.3 Data collection tools and methods

#### 2.3.1 Review of well‐being initiatives

Senior clinical staff and members of the education team were identified and invited in person or by email to answer questions about well‐being initiatives on the unit. Clinical members of the research team (RM, SW) were able to identify likely participants and data were gathered in person by clinical members of the team (RM, SW) or online (Zoom or Teams) by academic research team members (IB, OB) using a predesigned proforma (see Table 1).

**TABLE 1:** Well‐being initiatives proforma.

Item	Detail required
1	Background to the initiative
2	Perceived benefits of participating in this initiative
3	Details of how the initiative works in practice
4	Target population
5	Frequency of this initiative
6	Promotion of this initiative
7	Monitoring of participation
8	The uptake of this initiative
9	Measuring impact of this initiative

#### 2.3.2 Survey of awareness and uptake of well‐being initiatives

All clinical PCC staff employed at this study site were invited to take part in a brief survey via staff emails, closed social media pages and in person by clinical members of the research team (RM, SW) and/or unit administrative staff. The survey was available on paper and online (using Qualtrics). Paper copies were returned to the PCC research office, scanned and transferred to the host university's secure cloud storage. Surveys were anonymous, with just brief demographic data collected to describe the sample.

### 2.4 Data analysis

#### 2.4.1 Review of well‐being initiatives

Proformas (see Table 1) were used to collect details of existing initiatives.

The detailed descriptions of each intervention were then used to conduct BCT coding to identify ‘active ingredients’ of each initiative, informed by the BCT Taxonomy.^26^ This analysis was conducted by two academic researchers who had completed online BCT training: one researcher (IB) coded all initiatives identified and another (OB) coded 25% independently. Any discrepancies were resolved through discussion. The following assumptions guided the BCT coding analysis:

1. All initiatives were coded with the assumption that BCTs operated through enabling an individual to maintain an optimal level of well‐being. That is every initiative was perceived as helping staff in PCC to achieve good well‐being.
2. If specific reasons behind the BCTs were unclear, BCTs were coded at a general information level using what was available.
3. When initiatives were lacking in information and detail, the initiative lead was contacted for further details.
4. No initiative was discounted on the basis that there was not enough information to code.
5. Equal value and weighting were given to the 16 BCT groups and the 93 hierarchically clustered techniques. Findings are reported at the BCT group level.

#### 2.4.2 Survey of awareness and uptake of well‐being initiatives

Anonymous survey responses were subject to frequency analyses to determine the numbers of staff who were aware of and accessed the well‐being initiatives identified.

#### 2.4.3 Behaviour change wheel intervention build

The steps described in the BCW intervention development framework (see Figure 1) were undertaken to build the ‘SWell’ staff well‐being interventions (led by RLS with clinical expertise from HPD).^23^

### 2.5 Ethical and institutional approvals

Funding was received from Birmingham Women's and Children's NHS Foundation Trust Paediatric Intensive Care charities (37‐6‐124). Ethical approval was obtained from the Health Research Authority (HRA) on 27 August 2020 (20/HRA/3817) and Aston University on 22 June 2020 (UREC280719). Capability and capacity approval was obtained from Research and Development department at the NHS Trust.

### 2.6 Public and patient involvement and engagement (PPIE)

A national public and patient involvement and engagement (PPIE) group was established in collaboration with the paediatric critical care society (PCCS) Well‐being Special Interest Group involving PCC consultant intensivists, PCC Advanced Nurse Practitioners, educators and nurses. This group holds regular meetings which facilitated discussions with PPIE representatives on the following topics: research objectives, ideas for intervention development, factors to consider for implementation, and preliminary findings. Findings were also presented at national PCCS conferences to gain additional feedback from delegates. PPIE members' recommendations provided invaluable clinical expertise particularly in relation to how staff engagement with interventions could be facilitated on busy units.

### 2.7 Reflexivity

The research team was made up of clinicians who worked in the PCC unit where data were collected and by independent university researchers. The team shared their beliefs about well‐being and assumptions about what they thought would be successful prior to embarking on the study. During each team meeting and at every design decision point, the team were cautious to ensure decisions were led by what was required by the research and not by any individual's own biases. All data collected were anonymous. When potential participants were approached by clinical team members in person it was made clear that they could decline and/or complete their survey online thereby submitting their data directly to the university and bypassing clinical colleagues. (NB. all data collected were anonymous; no personal details were required).

## 3 RESULTS

### 3.1 Review of well‐being initiatives

**FIGURE 2:**
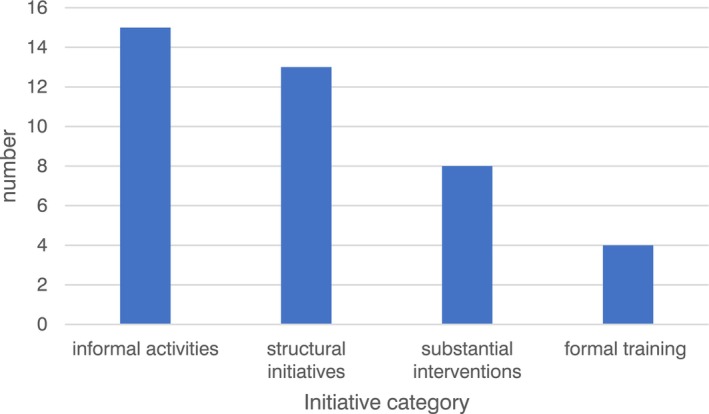
Well‐being initiative by category.

**FIGURE 3:**
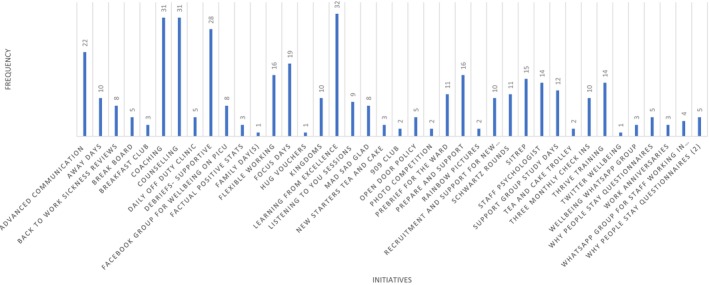
Frequency of behaviour change techniques (BCTs) across 40 well‐being initiatives identified.

In total, 40 initiatives were identified (see Supplementary File S1) and categorized into *informal activities*, for example, breakfast competition, and initiatives set up during the COVID‐19 pandemic to provide support during that time, for example, hug vouchers; longer‐standing *structural initiatives*, for example, small peer support groups named ‘kingdoms’, flexible working, back to work sickness reviews; to more *substantial interventions*, for example, introducing a staff psychologist, supportive debriefs; and *formal training*, for example, advanced communication training. (See Figure 2 for categorization of well‐being initiatives and Figure 3 for frequencies of BCTs in each initiative).

The most commonly used BCTs within the existing well‐being initiatives were defined in the BCT taxonomy as (see Table 2):

- Social support to provide support from peers and colleagues (identified in 32 initiatives);
- Self‐belief to boost self‐confidence and belief in one's ability to take control of a situation through reflection, mindfully practising new skills and building on previous success (in 28 initiatives);
- Goals and planning to raise awareness of threats to one's well‐being and to identify explicit, goal‐defined strategies with the intended outcome of improving one's well‐being (28 initiatives);
- Feedback and monitoring to observe and record what goes well and what could be improved, providing clear feedback enabling staff to monitor outcomes of their strategies to improve their well‐being (19 initiatives); and
- Shaping knowledge to improve staff understanding of what well‐being means to them, how it might be threatened, to equip them with successful strategies for improving and maintaining their well‐being (15 initiatives).

**TABLE 2:** Psychologically informed well‐being initiatives.

Name	Behaviour change technique groups	Description
Advanced communication training (Formal training)	‐ Goals and planning ‐ Feedback and monitoring ‐ Shaping knowledge ‐ Social support ‐ Natural consequences ‐ Comparison of behaviour ‐ Repetition and substitution ‐ Identity	Insufficient communication contributes to increased stress, lack of work satisfaction and emotional burnout. This training is designed to build staff confidence in their ability to communicate with patients' parents in challenging situations. Actors are used in simulated scenarios. Confidence levels are measured pre‐ and post‐training.
Supportive debriefs (Substantial intervention)	‐ Goals and planning ‐ Feedback andmonitoring ‐ Social support ‐ Shaping knowledge ‐ Natural consequences ‐ Comparison of behaviour ‐ Associations ‐ Comparison of outcomes ‐ Regulation ‐ Antecedents ‐ Self‐belief	This is an evidence‐based, psychologically informed debrief designed to share and normalize feelings following cardiac arrests and deaths. A ‘hot’ debrief is held immediately after an event and then an after‐action review is held around 1 h afterwards. Supportive debriefs are held a few days later.
Kingdoms (Structural initiative)	‐ Goals and planning ‐ Feedback and monitoring ‐ Social support ‐ Self‐belief	Kingdoms are small peer support groups which rotate every 4 weeks. They create a ‘home’ for nurses, clinical support workers and health care assistants providing a smaller group of staff they can get to know well.
Learning from Excellence (Substantial intervention)	‐ Goals and planning ‐ Feedback and monitoring ‐ Social support ‐ Shaping knowledge ‐ Natural consequences ‐ Comparison of behaviour ‐ Repetition and substitution ‐ Comparison of outcomes ‐ Reward and threat ‐ Antecedents ‐ Identity ‐ Self‐belief	This is a social movement predicated on a positive psychology approach rather than the detriment model often used in safety research which focuses on learning from mistakes. Learning from excellence prioritizes learning from positive feedback. A positive reporting system has been introduced for staff to report excellence when it is witnessed. Research has demonstrated positive impact on quality, safety, staff motivation and performance.^24^, ^26^
Prepare‐Support (Substantial intervention)	‐ Goals and planning ‐ Feedback and monitoring ‐ Social support ‐ Shaping knowledge ‐ Associations ‐ Regulation ‐ Self‐belief	This is an evidence‐based, theoretically informed simulation training session designed to psychologically prepare staff for caring for children when they experience an acute life‐threatening event.^25^, ^27^ Actors are used in scenarios focused on communication and teamwork to build self‐efficacy, competence and resilience for real‐life events.
PICU staff psychologist (Substantial intervention)	‐ Goals and planning ‐ Feedback and monitoring ‐ Social support ‐ Antecedents ‐ Regulation	A dedicated psychologist position was created to provide psychological support to staff. The service is developed based on a tiered approach of universal support, targeted support and clinical treatment to build a foundation of psychological safety in the unit with one‐to‐one support available for those who need it.^19^, ^26^, ^28^

The well‐being initiatives with the clearest usage of these psychologically informed BCTs included formal training, structural initiatives and substantial interventions.

### 3.2 Survey of awareness and uptake of well‐being initiatives

A mixed discipline sample of staff (*n* = 163) in the host PCC unit completed the survey of awareness and uptake of well‐being initiatives. Of the psychologically informed initiatives (see Table 2), *Supportive debriefs* had the highest levels of awareness (96%) and engagement (79%) within the sample, followed by *Learning from Excellence* (89% and 77%), *Kingdoms* (70% and 69%), *Prepare‐Support* (55% and 55%), *Advanced Communications Training* (67% and 51%) and *PICU staff psychologist* (56% and 27%).

Of those who completed the survey, 55% felt they were sometimes able to access well‐being initiatives that met their needs, 19% frequently and 18% rarely demonstrating that staff needs were not currently being met. Barriers to access included prioritizing family time instead of attending well‐being sessions on days off, feeling overwhelmed by work and not being on social media outlets where initiatives were advertised. Indeed, 69% reported they simply did not have time.

This review and BCT coding of existing well‐being initiatives was presented to the PPIE group at PCCS Well‐being Special Interest Group meetings. They were keen to ensure the interventions would bring staff together, work sensitively to help them understand their own well‐being, for them to improve staff's emotional literacy and enable them to recognize their own stressors to help manage them before they reached crisis point. They were very instructive in their emphasis on the interventions being as low‐resource as possible both in terms of cost and time.

The survey findings resonated with the PPIE perspective and again emphasized the need for staff well‐being interventions to be as low‐resource (in relation to training and time required for delivery and financial cost of delivery and backfill for staff attendance) as possible and for them to be advertised openly without reliance on social media. It is true that those informed by psychological theory and/or evidence were also the most time‐intensive, larger‐scale interventions. While these interventions meet a need, it is a specialized need, in terms of psychological qualifications and/or advanced level of expertise required to deliver them. It was clear from poor uptake of existing initiatives and the PPIE feedback that there was a need to build something more generic which could be employed more generally and more flexibly across different groups of staff.

### 3.3 New empirical evidence

Several studies have been undertaken by the wider *SWell* project team to explore PCC staff's own ‘lived experience descriptions’ of well‐being, that is, what well‐being means to them, prerequisites for good workplace well‐being, that is, what is needed for well‐being to exist and the nature of workplace challenges to their well‐being. These studies add to the literature reviewed above.

One study with multidisciplinary PCC staff supported the Australian study^18^ above, using Appreciative Inquiry^28^ to identify the constituent elements of well‐being as: being nurtured and supported at work, being in nature and social support independent from work.^29^ The importance of feeling nurtured at work was confirmed by two other studies, one with newly qualified nurses^1^ and another with more experienced nurses and allied health professionals.^30^ These studies showed the importance of being able to express vulnerabilities with colleagues and thereby access peer support, both inside and outside of work. Those studies identified that PCC staff struggled to establish work‐life balance and often their psychological distress would leak into their home life. Some staff had established ways of managing the pressures of working in PCC, but others expressed a lack of confidence in their ability to identify and engage in activities which would boost their well‐being.^1^, ^30^

These findings support theoretical evidence that a sense of belonging is a prerequisite for workplace well‐being.^10^, ^31^ This suggests that a group intervention could help build peer‐to‐peer relationships and therefore foster well‐being. Alongside that, the intervention needs to build individuals' confidence in managing their well‐being. This could be achieved within the group setting with more confident individuals sharing what works for them.

Two other studies with PCC nurses and doctors^32^, ^33^ supported previous research^18^ in identifying critical treatment decisions and moral distress as significant challenges to workplace well‐being. This further supports the need to create psychologically safe environments where staff are able to share their personal experiences in a non‐judgemental space.^21^, ^34^ For the group context to be conducive to fostering well‐being, therefore, it needs to be run sensitively by well‐equipped staff and made up of staff groupings willing to share personal experiences with each other.

In addition, PCC consultants were keen to convey that some hospital initiatives, such as organizing yoga sessions were inappropriate, inaccessible and tokenistic.^32^ This points towards a more equitable approach to well‐being interventions which are available to all shift patterns and which tackle the particular challenges that have been identified as important to PCC staff.

### 3.4 Behaviour change wheel intervention build (steps 1–4)

**FIGURE 4:**
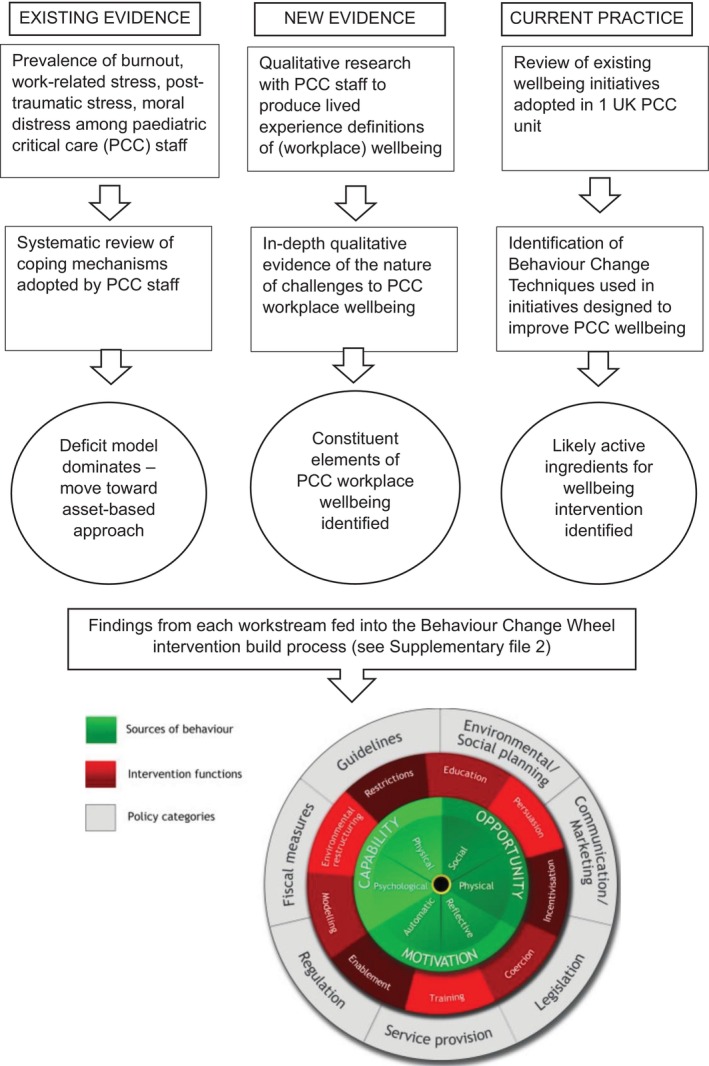
Flow diagram of *S*taff *Well*being (SWell) intervention development. Behaviour Change Wheel diagram reproduced with permission from: Michie S, Atkins L, West R. The Behaviour Change Wheel: a guide to designing interventions. London: Silverback Publishing; 2015.

A subgroup of the research team (led by RLS, with clinical expertise from HD, SW) then worked through the BCW steps using evidence from the well‐being initiative review and survey, the PPIE advice and the new empirical evidence see Figure 1.^1^, ^29^, ^32^, ^33^ (See Figure 4 for a summary of how these phases of work were combined to build the *SWell* interventions).

#### 3.4.1 Step 1: Behavioural analysis

Behavioural analysis identified the target behaviour as improved self‐care with the primary outcome measure of improved well‐being. The Short Warwick‐Edinburgh Mental Well‐Being Scale^35^ would provide a standardized measure of well‐being pre‐ and post‐intervention. Appropriate standard measures for secondary outcomes in a test of effectiveness would be resilience (using the Brief Resilience Scale)^36^ and anxiety and depression (using the Hospital Anxiety and Depression Scale)^37^ (NB. The test of effectiveness is out of the scope of this paper).

#### 3.4.2 Step 2: Capability‐opportunity‐motivation determinants of behaviour change

The Capability‐Opportunity‐Motivation model of Behaviour (COM‐B)^23^ is a theory which identifies the determinants of behaviour as capability, opportunity and motivation. This means the intervention needs to aim to:

- Build staff's *capability* to engage in well‐being‐supportive activities
- Create the *opportunity* for staff to attend well‐being intervention sessions and
- Ensure staff are *motivated* to change their behaviour in the short‐term but also to commit to well‐being‐supportive behaviours to develop new habits.

(See Supplementary File S2 for a diagram which describes this COM‐B analysis in detail).

#### 3.4.3 Step 3: Identification of appropriate intervention function(s)

A set of intervention functions is included in the BCW framework (see Figure 1) which can be used when developing new interventions. Based on the work undertaken thus far, the functions identified as most appropriate for PCC staff well‐being interventions were:

- Modelling—likely to be beneficial given the significance of team identity and belonging in the lived experience definitions of workplace well‐being^29^, ^30^; seeing ‘someone like me’ engage in well‐being‐supportive, self‐care behaviours is likely to increase readiness to change and adoption of self‐care behaviours.
- Enablement—important to give staff sanctioned time away from the unit to engage in the well‐being intervention.^32^
- Persuasion—in the form of prompts to encourage discussion would be a useful function, especially given the difficulty of defining well‐being.^29^
- Education—training staff to name and process their emotions; and to become self‐aware of their own individual stressors and what helps and hinders those stressors.
- Training—in terms of developing individualized self‐care skills.

#### 3.4.4 Step 4: APEASE assessment of feasibility

The final BCW step is using what is known as the APEASE criteria^23^ to identify how to make the intervention feasible. This is achieved by assessing the following:

- Acceptability—the intervention needs to be perceived by staff as worthwhile
- Practicability—it needs to fit within shift patterns
- Effectiveness—it needs to be judged with a measure of well‐being (e.g., Warwick‐Edinburgh Mental Well‐Being Scale, as identified above)
- Affordability—the intervention needs to be delivered in‐house by staff who do not need specialist psychological training.
- Spillover effects are unintended consequences, which need to be explored when the intervention is piloted.
- Equity—requires the intervention to be available to all staff.

### 3.5 The ‘SWell’ staff well‐being interventions

While the psychologically informed interventions listed in Table 2 featured evidence‐based BCTs, they were too time‐consuming, costly and required specialist training which made them infeasible following the APEASE assessment of feasibility. This meant it was necessary to return to the full set of initiatives to identify any that met the requirements identified in the BCW process which would also meet the APEASE criteria for feasibility.

#### 3.5.1 Mad‐Sad‐Glad retrospective exercise

A version of the Mad‐Sad‐Glad intervention^38^ was identified from the review as a candidate for feasibility testing. It was categorized as a substantial intervention, but is run with small groups of staff, is flexible in terms of length of sessions and requires minimal training for staff who are used to facilitating group discussions. At the time, Mad‐Sad‐Glad was novel to PCC and the study site, but was developed from the business field as a retrospective reflective group exercise involving identification of situations at work which make staff feel mad, sad and glad.^38^ It works through a series of questions to guide them through the steps of naming and processing those feelings and their impact on their well‐being. The discussion focuses on feelings in a non‐judgemental space without apportioning blame.

BCTs in Mad‐Sad‐Glad are: social support, self‐belief and feedback and monitoring. These were identified in the review of existing initiatives as likely to be most suited for PCC staff well‐being intervention. Summary descriptions of these BCTs and how they are implemented in the ‘Swell’ interventions are presented in Table 3. The functions used in Mad‐Sad‐Glad are presented in Table 4.

**TABLE 3:** Behaviour change techniques (BCTs) in Mad‐Sad‐Glad and wellbeing images interventions.

BCTs	Description	Implementation
Social support	To provide support to peers, colleagues, friends, family	To bring staff together to share experiences so they can provide social support for each other
Self‐belief	To boost self‐confidence and one's belief in one's ability to take control of a situation through reflection, practising new skills and building on previous successes	To build confidence in staff's ability to identify and express their emotions in response to work
Feedback and monitoring	To observe and record what goes well and what could be improved to provide clear feedback enabling staff to monitor the outcomes of their strategies	To encourage staff to monitor what increases their stress, when they experience challenging emotions, what helps boost their well‐being

**TABLE 4:** Functions in Mad‐Sad‐Glad and wellbeing images interventions.

Intervention function	Description	Implementation
Modelling	Providing an example for people to aspire to or imitate	Creating exemplar case studies to promote self‐care, identify influential staff to model healthy self‐care behaviours
Persuasion	Using communication to induce positive or negative feelings or stimulate action	Prompts (communication, images) to elicit postive feelings of well‐being
Enablement	Increasing means/reducing barriers to increase capability or opportunity	Ensuring attendance at intervention sessions is endorsed and accommodates shift patterns
Education	Increasing knowledge or understanding	Providing information related to self‐care and well‐being, enabling staff to identify and name their emotions to help them process them
Training	Imparting skills	Raising awareness of psychological skills for: self‐care, identifying stressors, naming and processing emotions, identifying one's stress relief and well‐being‐supportive techniques

(See Supplementary File S3 for the TIDieR (Template for Intervention Description and Replication) intervention checklist, which is a tool used to ensure the features of an intervention are reported transparently to facilitate replication).^39^

#### 3.5.2 Well‐being images with appreciative inquiry

Mad‐Sad‐Glad focused explicitly on challenges to workplace well‐being, but alongside that, the empirical evidence demonstrated the significance of work‐life balance and individuals' subjective meanings of and ways of managing well‐being. As a result, it was important to offer an intervention with a more holistic approach to well‐being, which would help staff articulate what well‐being meant to them. There was nothing which did this explicitly in the interventions reviewed. The research team reflected on methods in the empirical work cited above, in particular two studies which used Appreciative Inquiry^28^ and a set of images to explore with PCC staff what represented well‐being to them.^29^, ^30^

The images of scenery, seascapes, children, animals, musical instruments, friends were shown to staff and they were asked to identify one which represented well‐being to them. Appreciative Inquiry^28^ was used to encourage open‐minded, imaginative discussion in four phases: *discovery*—to explore what well‐being means; *dream*—identifying wishes to improve well‐being; *design*—determine goals for improving well‐being; and *destiny*—making it happen by identifying how to overcome challenges and identifying support required. Appreciative Inquiry requires a non‐judgemental environment to ensure staff feel comfortable sharing their personal experiences, which meets the requirement that the intervention is conducted in a psychologically safe space.

The BCTs used in Well‐being Images are: social support, self‐belief and feedback and monitoring (see Table 3). Its functions are listed in Table 4. The function used in Well‐being Images are included in Table 4. (See Supplementary File S4 for the TIDieR checklist).

Both Mad‐Sad‐Glad and Well‐being Images were presented to the PPIE group (PCCS Well‐being Specialist Interest Group) and other stakeholder groups, for example, Operational Delivery Networks for PCC nursing and NHS England Children and Young People's Nursing Directorate. Feedback was used to further refine the interventions and to identify skills required to facilitate the sessions. All the learning from the BCW intervention development work will inform the next phase of the project, which is to conduct a feasibility study in UK PCC units (reported separately).

## 4 DISCUSSION

This study successfully developed two ‘SWell’ (*S*taff *Well*being) interventions using the systematic BCW framework and specified psychological BCTs, informed by empirical evidence from research with PCC staff.

The key issues highlighted by the BCW process were that the PCC setting poses key resource challenges, chiefly time and cost. Although the review of existing well‐being initiatives identified some highly specialized, evidence‐based and psychologically informed interventions and training programmes, it would be impossible to test their feasibility as a generic intervention to improve staff well‐being without significant investment. They simply required too much specialist knowledge, were costly and too time‐intensive to be rolled out nationwide.

Findings from the survey of awareness and uptake of well‐being initiatives chimed well with PPIE feedback emphasizing the need for something flexible. Furthermore, for the interventions to have the chance of becoming part of standard practice within PCC units, they needed to be flexible to the local context, where shift patterns, workload, workforce size and/or skill mix might require adaptation for successful delivery.

Together, the review of existing well‐being initiatives and the new empirical evidence presented identified the BCTs that would be the focus of the SWell interventions. The ensuing discussion reflects upon the choices of BCTs, the ‘active ingredients’ of the interventions with reference to wider literature and psychological theory.

Following the review of initiatives, awareness survey and the BCW process, informed decisions were made for the interventions to target self‐belief (BCTs are presented in bold and theoretical concepts in italics.) and confidence—or *self‐efficacy* ^40^—to improve staff's ability to recognize what well‐being means to them and to notice when they experience challenges to their workplace well‐being. This positive focus aligns with the self‐determination theory of thriving,^31^ which has been adopted by some in health care to identify the requirements for workplace well‐being.^10^ This focus on psychological theory meant the SWell interventions were designed specifically to create feelings of *autonomy* and *competence*, both factors identified which help individuals to thrive.

Through their social support (or peer support), the SWell interventions of Mad‐Sad‐Glad and Wellbeing Images encouraged a sense of *belonging* or *relatedness* (from the self‐determination theory), which the empirical work highlighted as central to workplace well‐being; something highlighted even further when staff were redeployed to other (adult) critical care units during the COVID‐19 pandemic with unfamiliar people, places and systems.^1^, ^29^, ^30^ The power of team identity and the positive experience of teamworking when members come together like ‘a big crazy, chaotic orchestra’ was described as ‘phenomenal’ and central to workplace well‐being^29^, ^33^ which made that group, peer‐to‐peer aspect of the SWell interventions significant.

The empirical evidence which informed the intervention development had identified the importance of *psychological safety* when working to explore and improve workplace well‐being.^21^ It was clear that staff could experience significant vulnerabilities through their everyday practice in PCC^1^, ^18^, ^30^, ^33^ and if they encountered critical incidents which required investigation, that vulnerability could have a life‐ or career‐altering impact on PCC staff.^22^ It was therefore crucial to ensure that staff felt safe to express their emotions and that they trusted each other. This would have implications for the make‐up of groups invited to SWell intervention sessions. Furthermore, it made clear that the facilitator of SWell interventions would need to understand the psychological theory underpinning Mad‐Sad‐Glad and Wellbeing Images and that they were skilled in facilitating group discussions in an open, honest and fair manner.

Finally, the importance of acknowledging the coexistence of both positive well‐being and threats to well‐being at work was clear.^18^, ^33^ This required the intervention to focus on self‐awareness and developing *reflective motivation* to enable staff to practise self‐care, that is, the target behaviour to change. This was achieved in the use of feedback and monitoring as a BCT within the group session itself. Ideally, though, to benefit fully from feedback and monitoring—or *self‐regulation* ^41^—individuals attending SWell interventions would be encouraged to intermittently monitor any changes in the target behaviour of self‐care that they made so the (in)direct impact on their sense of well‐being would be consciously noted. This feedback and monitoring—or *self‐regulation*—creates the link between target behaviour (self‐care) and outcome (well‐being), and is the mechanism by which change is first noticed and maintained over time.

### 4.1 Limitations

This study took place in one large PCC unit in England. It is not necessarily representative of all 28 UK PCC units, but national and international research demonstrates that experiences are likely to resonate strongly in other units and countries.^1^, ^2^, ^5^, ^6^, ^30^, ^32^, ^42^ Nevertheless, future research examining interventions to improve PCC well‐being worldwide would be beneficial.

It is important to note that the SWell interventions developed are group‐based, largely to increase the feasibility of delivering them in busy PCC units. Nevertheless, they are designed to enable individuals to work in the group to identify their own understanding of well‐being and their idiosyncratic strategies for managing their well‐being. Some staff may still require or prefer an individual session. Further research is required to design and operationalize individual interventions, such as one‐to‐one well‐being conversations.

While the SWell interventions are built from existing methods and techniques,^28^, ^38^, ^43^ they are original in the following ways: (i) they are the first bespoke staff well‐being interventions built for the PCC environment, (ii) based on psychological theory and (iii) contemporary evidence (iv) using a systematic model of intervention development^44^ which aligns closely with gold standard guidelines for the development and evaluation of complex behavioural interventions.^25^, ^45^ As such, this project has produced a new set of *SWell* (*S*taff *Well*being) interventions that are highly likely to be feasible in the pressured environment of PCC that will contribute to building a workplace environment that prioritizes staff well‐being.

### 4.2 Clinical implications and recommendations for future research

The implications of this work hold significant promise of far‐reaching improvement in staff well‐being. The SWell interventions have been designed to be flexible, deliverable by in‐house staff, to fit within shift patterns, to provide a low‐intensity intervention to improve staff well‐being. The psychological impact on individual staff and the workforce as a whole could contribute to the creation of compassionate work environments which prioritize psychological safety where well‐being becomes the norm.^21^, ^34^ The potential impact of creating a thriving workforce is a reduction in staff attrition, sickness absence and ultimately better quality care for patients.^10^, ^12^, ^14^

## 5 CONCLUSION

The improvement of staff well‐being does not happen in isolation and there are other significant factors requiring attention in UK health care for real change to occur and be maintained. Investment in staff well‐being is required both in terms of time for staff to deliver and attend well‐being interventions but also in terms of the organizational worth that is placed on the psychological well‐being of the health care workforce. Confidence in the likely success of the ‘SWell’ interventions comes from the fact that they are evidence‐based, psychologically informed and designed specifically for PCC units. This increases their likelihood of being feasible and could have a significant and positive impact on workforce well‐being.

## AUTHOR CONTRIBUTIONS

Rachel Shaw was chief investigator, responsible for conceptualization of the project, led funding acquisition, managed the project, line‐managed research associates, oversaw data curation and data analysis and led the writing of the original manuscript draft. Isabelle Butcher was postdoctoral research associate, she led day‐to‐day participant recruitment, data collection and analysis and supervised a research assistant during the review of existing well‐being initiatives and Behaviour Change Techniques (BCTs) coding. She contributed editorially and has approved the final version of the manuscript. Sarah Webb was co‐applicant in funding acquisition, contributed to conceptualization of the project, recruitment and data collection for the review of well‐being initiatives and led on refinement of Mad‐Sad‐Glad. She reviewed and has approved the final version of the manuscript. Heather Duncan was co‐applicant in funding acquisition, contributed to conceptualization of the project, provided clinical expertise which informed the Behaviour Change Wheel process, reviewed, edited and approved the final version of the manuscript. Rachael Morrison was principal investigator at the study site and co‐applicant on funding acquisition. She contributed to conceptualization of the project, recruitment and data collection of the review of well‐being initiatives, contributed to BCT coding and reviewed and approved these aspects of the final version of the manuscript.

## FUNDING INFORMATION

This project was funded by Birmingham Women's and Children's NHS Foundation Trust Paediatric Intensive Care charities (37‐6‐124).

## CONFLICT OF INTEREST STATEMENT

The authors declare no conflicts of interest.

## PATIENT CONSENT STATEMENT

All participants gave informed consent.

## Supporting information


**Data S1.** Supporting Information.

## Data Availability

Research data are not shared.
