# Supplementary material for: Building evidence‐based interventions to improve staff well‐being in paediatric critical care using the behaviour change wheel
Source: Nurs Crit Care. 2025 Jan 8;30(4):e13228. doi: 10.1111/nicc.13228 (PMC12210092; doi:10.1111/nicc.13228)
Supplement: Supplementary file 1 — Data S1. Supporting Information. [file NICC-30-0-s001.docx]

# Supplementary file 1: Wellbeing initiatives identified

| Advanced communications  ALL STAFF | Away Days  CONSULTANTS, BAND 7-8,  ANP BAND 6 | Back to work sickness reviews NURSES, ANP | Break board BAND 5-6 | Breakfast club MEDICAL TRAINEES |
| --- | --- | --- | --- | --- |
| Coaching ALL STAFF | Counselling ALL STAFF | Daily off duty clinic NURSES | Supportive debriefs ALL STAFF | Facebook group for wellbeing on PICU  ALL STAFF |
| Factual positive stats  ALL STAFF | Family day(s)  ALL STAFF | Flexible working  ALL STAFF | Focus days ALL STAFF | Hug vouchers  ALL STAFF |
| Kingdoms  NURSES, CSW, HCA | Learning from excellence  ALL STAFF | Listening to You sessions  ALL STAFF | Mad Sad Glad sessions BAND 5 | New starters Tea and Cake  BAND 5 |
| Nine 09 zoom club  ALL STAFF | Open door policy  NURSES | Photo competition  ALL STAFF | Pre brief for the ward ALL STAFF | Prepare and Support  ALL STAFF |
| Rainbow pictures ALL STAFF | Recruitment and support for new international trainees  TRAINEES | Schwartz rounds ALL STAFF | SITUATIONAL REPORT ALL STAFF | Specialised Staff psychologist (PICU)  ALL STAFF |
| Support group study days  NURSES | Tea and cake trolley ALL STAFF | Three-month check-ins ALL STAFF | Thrive training BAND 5 | Twitter wellbeing ALL STAFF |
| Wellbeing group ALL STAFF | Why people stay questionnaires  ALL STAFF | Work anniversaries MISSING | WhatsApp support group ALL STAFF | Why people stay questionnaires – interviews  DUPLICATION |
| **Key:** | **Informal activities** | **Structural initiatives** | **Substantial interventions** | **Formal training** |

For further information contact the corresponding author: [r.l.shaw@aston.ac.uk](mailto:r.l.shaw@aston.ac.uk)

**Motivation**

**Social opportunity**

**Reflective motivation**

**Automatic motivation**

**Opportunity**

**Physical opportunity**

**Physical opportunity:** space, time & resources to work on wellbeing/self-care.

**Social opportunity:** employer recognition of importance of wellbeing, acceptance of self-care among colleagues, sense of belonging within working team.

**Reflective motivation:** consciously contemplate: wellbeing, challenges to wellbeing, beliefs that wellbeing can be improved successfully, how to enact self-care successfully.

**Automatic motivation:** prioritising wellbeing to become habitual, everyday activity within workplace (& homelife). Staff enjoy improving their wellbeing and reap the benefits

Behaviour

**Changed self- care behaviours to improve wellbeing**

# Supplementary file 2: COM-B Behavioural analysis to develop Swell interventions to improve staff wellbeing

**Capability**

**Physical capability**

**Psychological capability**

**Physical capability:** enact wellbeing-supportive activities (e.g., in/outdoor activities, relaxation, talking, meditation).

**Psychological capability:** psychological skills, self-awareness (of signs of stress & key stressors, own subjective wellbeing, knowledge of what helps/hinders wellbeing).

**Supplementary file 3: TIDieR checklist - *Mad-Sad-Glad Retrospective Exercise***

The format of this group exercise was taken from TeamRetro[1](#_bookmark0), an organisation providing group exercises to facilitate teamworking. It is an

exercise that had been adapted for new nurses working in PCC. We then refined it further for feasibility testing.

# Mad-Sad-Glad TIDieR Intervention Checklist

| ***Brief name*** | Provide the name or a phrase that describes the intervention | Mad-Sad-Glad |
| --- | --- | --- |
| ***Why*** | Describe any rationale, theory, or goal of the  elements essential to the intervention | This intervention asks staff to share what has recently made them feel frustrated or annoyed (mad), disappointed (sad) and what made them feel happy or proud (glad) while working. This enables staff to be more emotionally aware, which helps build a psychologically safe environment where sharing vulnerabilities is normalized.  The exercise provides an opportunity to reflect on issues and opportunities from different perspectives, and focuses the conversation on events, behaviours, and processes, not on assigning blame or ‘guilting’ individuals. |
| ***Who*** | Describe the target group(s) for this intervention | Newly qualified nurses were the target group when Mad-Sad- Glad was implemented at BCH. It could be the same group, and/or medical trainees, or indeed staff in any role with any  level of experience. |
| ***What*** | Materials: describe any physical or informational materials used in the intervention, including those provided to participants or used in intervention delivery or in training of intervention providers.  Provide information on where the materials can be  accessed (such as online appendix, URL) | Behaviour Change Techniques:   - Social support - Self-belief - Feedback & monitoring   Materials: |

^1^ [www.teamretro.com/retrospectives/mad-sad-glad-retrospective](http://www.teamretro.com/retrospectives/mad-sad-glad-retrospective)

|  |  | - Coloured sticky notes (3 different colours for either mad, sad or glad), pens, flipchart (or electronic alternatives   Procedures:   - Gather the group together (in a peer/support group meeting of newly qualified staff, or with other staff groups it could follow an existing meeting, or a meeting specially formed for this purpose). - Introduce the goals and parameters of the exercise. - Ask staff to individually list the following using the coloured sticky notes:   - Mad: list the things that annoy you most. How does this affect your work?   - Sad: What causes grief, disappointment or sorrow? What would help you manage (or process) those things well?   - Glad: What makes you happy when you think about your work? What are the elements that you enjoy the most?   Icebreakers: e.g., right now, are you mad, sad, or glad? Would you rather be sad or mad? What’s made you feel glad recently?   - Group discussion: collate the sticky notes onto a flipchart/board, identify common themes and then the group votes which they’d like to discuss in more depth in order of importance. - Action: the group are asked what actions they could take to improve those things that make them mad or sad and how they can share the joy of what makes them   glad. |
| --- | --- | --- |
| ***Who provided*** | For each category of intervention provider (such as psychologist, nursing assistant), describe their | - No formal qualifications are required; however the facilitator will need to be skilled in group facilitation and have a good understanding of Mad-Sad-Glad. |

|  | expertise, background, and any specific training  given | - Training will include:   - Background to the SWell project   - Development of the SWell Interventions using   health psychology theory   - - Delivering Mad-Sad-Glad |
| --- | --- | --- |
| ***How*** | Describe the modes of delivery (such as face to face or by some other mechanism, such as internet or telephone) of the intervention and whether it was  provided individually or in a group | - Small peer groups of approx. 10 staff members - Newly qualified staff groups held at 3, 6, and 9 month intervals (or at appropriate intervals linking to existing staff meetings or catch-ups) |
| ***Where*** | Describe the type(s) of location(s) where the intervention occurred, including any necessary infrastructure or relevant features | - Private room (capacity approx. 10) or zoom/teams meeting (ideally better in person) |
| ***When and how much*** | Describe the number of times the intervention was delivered and over what period of time including the number of sessions, their schedule, and their duration, intensity or dose | - Attendance is voluntary but encouraged - Sessions should last approx. 45 minutes to an hour (but could be shorter with tight facilitation and ‘quick fire’ responses) |
| ***Tailoring*** | If the intervention was planned to be personalised,  titrated or adapted, then describe what, why, when,  and how | - Invitations to staff groups could be extended beyond newly qualified staff - Repeat sessions may not be possible within time frame |
| ***Modifications**** | If the intervention was modified during the course of the study, describe the changes (what, why, when, and how) | - All staff groups were invited - Sessions usually happened within an existing study day - Attendance was one-off rather than at repeated intervals |
| ***How well*** | Planned: If the intervention adherence or fidelity was assessed, describe how and by whom, and if any strategies were used to maintain or improve fidelity, describe them | - Feasibility and acceptability questions (Likert scale ratings and open-ended questions) were asked of intervention attendees via online survey immediately after intervention sessions. - Feedback was collected from staff delivering the   intervention at the end of the intervention period |
| ***How well*** | Actual: If intervention adherence or fidelity was assessed, described the extent to which the intervention was delivered as planned | - As above - In addition, qualitative feedback on delivery was received at study events throughout the course of the project. |

* If checklist is completed for a protocol, these items are not relevant to protocol and cannot be described until study is complete.

**Supplementary file 4 – TIDieR Checklist – *Wellbeing Images with Appreciative Inquiry***

| ***Brief name*** | Provide the name or a phrase that describes the intervention | Wellbeing Images with Appreciative Inquiry |
| --- | --- | --- |
| ***Why*** | Describe any rationale, theory, or goal of the elements essential to the intervention | Appreciative Inquiry is a well-known Quality Improvement method in healthcare. It encourages exploration and discussion around a particular topic through 4 stages:   - Discovery: appreciate the best of how things are now - Dream: imagine what could be - Design: determine what should be - Destiny: create what will be   Wellbeing is an abstract concept. This exercise prompts staff to discover what wellbeing means for them, both in work and outside of work. It does this by presenting a series image cards depicting a range of different images (e.g., green spaces, seaside, family, friends, animals, music) and asking them to choose one which represents wellbeing to them. The exercise then asks staff to dream about how wellbeing might be  improved and sustained in the future. |
| ***Who*** | Describe the target group(s) for this intervention | Any staff group or individual could be invited to take part in this intervention. It could be an existing group (peer group, support group) or a (random) group of willing volunteers pulled together  especially for this activity or as an add-on to an existing meeting. |
| ***What*** | Materials: describe any physical or informational materials used in the intervention, including those provided to participants or used in intervention delivery or in training of intervention providers.  Provide information on where the materials can be accessed (such as online appendix, URL) | Behaviour Change Techniques:   - Social support - Self-belief - Feedback & monitoring   Materials:   - Image cards representing different potential sources of wellbeing, pens, flipchart paper (or virtual alternatives) - Slides of images for online meetings |

|  |  | Procedures:   - Gather a group of staff together (at a peer/support group meeting, at a team meeting, in a special meeting facilitated for just this purpose). - Introduce the goals and parameters of the exercise. - Ask staff the following questions to work through the Appreciative Inquiry model:   - Discovery: pick an image that represents wellbeing to you. Tell us a bit more about it.   - Dream: What three wishes do you have to improve your wellbeing?   - Design: What would it take to create changes for your wellbeing at work?   - Destiny: What challenges might you encounter? How might you overcome them? What support would you need to achieve that? |
| --- | --- | --- |
| ***Who provided*** | For each category of intervention provider (such as psychologist, nursing assistant), describe their expertise, background, and any specific training given | - No formal qualifications are required; however the facilitator will need to be skilled in group facilitation and have a good understanding of Wellbeing Images with Appreciative Inquiry. - Training will include:   - Background to the SWell project   - Development of the SWell Interventions using health psychology theory   - Delivering Wellbeing Images   - Appreciative Inquiry background and delivery |
| ***How*** | Describe the modes of delivery (such as face to face or by some other mechanism, such as internet or telephone) of the intervention and whether it was  provided individually or in a group | - Small peer/support groups of 1 to 10 staff - Frequency is flexible and so can fit around other   meetings. The ideal would be to reach as many staff as possible in 6 months |
| ***Where*** | Describe the type(s) of location(s) where the intervention occurred, including any necessary infrastructure or relevant features | - Private room (capacity approx. 10) or zoom/teams room (ideally better in person) |

| ***When and how much*** | Describe the number of times the intervention was delivered and over what period of time including the number of sessions, their schedule, and their duration, intensity or dose | - Attendance is voluntary but encouraged - Sessions should last approx. 45 minutes to an hour (but could be shorter with tight facilitation and ‘quick fire’ responses) |
| --- | --- | --- |
| ***Tailoring*** | If the intervention was planned to be personalised,  titrated or adapted, then describe what, why, when, and how | - Ad hoc sessions could be delivered, e.g., in quiet  periods on nights, if wellbeing image cards are readily available |
| ***Modifications**** | If the intervention was modified during the course of the study, describe the changes (what, why, when, and how) | - Feasibility and acceptability questions (Likert scale ratings and open-ended questions) were asked of intervention attendees via online survey immediately after intervention sessions - Detailed feedback was collected from staff delivering   the intervention at the end of the intervention period |
| ***How well*** | Planned: If the intervention adherence or fidelity was assessed, describe how and by whom, and if any strategies were used to maintain or improve fidelity,  describe them | - As above - In addition, feedback on delivery was received at study events throughout the course of the project. |
| ***How well*** | Actual: If intervention adherence or fidelity was assessed, described the extent to which the intervention was delivered as planned | - Feasibility and acceptability questions (Likert scale ratings and open-ended questions) were asked of intervention attendees via online survey immediately after intervention sessions - Feedback was collected from staff delivering the   intervention at the end of the intervention period |

* If checklist is completed for a protocol, these items are not relevant to protocol and cannot be described until study is complete.
